# Implementation and Quality Control of Lung Cancer EGFR Genetic Testing by MALDI-TOF Mass Spectrometry in Taiwan Clinical Practice

**DOI:** 10.1038/srep30944

**Published:** 2016-08-02

**Authors:** Kang-Yi Su, Jau-Tsuen Kao, Bing-Ching Ho, Hsuan-Yu Chen, Gee-Cheng Chang, Chao-Chi Ho, Sung-Liang Yu

**Affiliations:** 1 Department of Clinical Laboratory Sciences and Medical Biotechnology, College of Medicine, National Taiwan University, Taipei, Taiwan; 2 Departments of Laboratory Medicine, National Taiwan University Hospital, Taipei, Taiwan; 3 Institute of Statistical Science, Academia Sinica, Taipei, Taiwan; 4 Division of Chest Medicine, Department of Internal Medicine, Taichung Veterans General Hospital, Taichung, Taiwan; 5 Faculty of Medicine, School of Medicine, National Yang-Ming University, Taipei, Taiwan; 6 Comprehensive Cancer Center, Taichung Veterans General Hospital, Taichung, Taiwan; 7 Department of Internal Medicine, National Taiwan University Hospital, Taipei, Taiwan; 8 Center of Genomic Medicine, National Taiwan University College of Medicine, Taipei, Taiwan; 9 Department of Pathology and Graduate Institute of Pathology, College of Medicine, National Taiwan University, Taipei, Taiwan; 10 Center for Optoelectronic Biomedicine, College of Medicine, National Taiwan University, Taipei, Taiwan

## Abstract

Molecular diagnostics in cancer pharmacogenomics is indispensable for making targeted therapy decisions especially in lung cancer. For routine clinical practice, the flexible testing platform and implemented quality system are important for failure rate and turnaround time (TAT) reduction. We established and validated the multiplex EGFR testing by MALDI-TOF MS according to ISO15189 regulation and CLIA recommendation in Taiwan. Totally 8,147 cases from Aug-2011 to Jul-2015 were assayed and statistical characteristics were reported. The intra-run precision of EGFR mutation frequency was CV 2.15% (L858R) and 2.77% (T790M); the inter-run precision was CV 3.50% (L858R) and 2.84% (T790M). Accuracy tests by consensus reference biomaterials showed 100% consistence with datasheet (public database). Both analytical sensitivity and specificity were 100% while taking Sanger sequencing as the gold-standard method for comparison. EGFR mutation frequency of peripheral blood mononuclear cell for reference range determination was 0.002 ± 0.016% (95% CI: 0.000–0.036) (L858R) and 0.292 ± 0.289% (95% CI: 0.000–0.871) (T790M). The average TAT was 4.5 working days and the failure rate was less than 0.1%. In conclusion, this study provides a comprehensive report of lung cancer EGFR mutation detection from platform establishment, method validation to clinical routine practice. It may be a reference model for molecular diagnostics in cancer pharmacogenomics.

The paradigm of lung cancer therapy has shifted from the histopathology-based to the companion diagnosis-based guidance since the emergence of TKIs (tyrosine kinase inhibitors) and immune check point therapy^1^. One of the important landmarks in this shift is the advance in discovery of tumor driver mutations. Tumor onset, progression and drug resistance are involved in altered signaling pathways that modulate the cancer hallmarks including tumor cell proliferation, motility, adhesion, angiogenesis and apoptosis and immune escape^2^. Molecules that can reverse or compensate the effects caused by these alterations have the potential to develop as anti-tumor drugs for molecular targeting therapy (MTT) or immune therapy. MTT has been proved as an efficient and better strategy to benefit cancer patients with actionable mutations in various cancers, especially in lung cancer^3^,^4^,^5^. Since the mutation burden was diverse in different cancer types, certain genetic aberrations, so-called driver mutations, led tumor cells to depend on or addict to a mutation-dependent signaling pathway^6^,^7^. Hence, the identification of these driver mutations is necessary for the treatment decision. To identify patients who benefit from MTT by molecular testing is one of the most important issues of precision medicine. In lung cancer, the leading cause of cancer-related death worldwide^8^, the molecular testing-based target therapy has been routinely practiced. Recently, Kris *et al.* showed that patients with driver mutations who received the corresponding drugs had a prolonged progress-free survival than those with a driver mutation who did not receive the drugs and those without driver mutations^9^. This suggested that the companion molecular diagnostics-guided therapy is the trend in cancer management to improve patients’ survival^10^.

However, several critical issues that should be concerned including reliability, reproducibility, specimen amount, sample quality and turnaround time before cancer molecular testing become routine assays. Until now, many home-made and commercialized methods have been utilized for detecting the specific cancer associated gene mutations, such as EGFR mutations in lung cancer measured by Sanger sequencing, PCR-SSCP (single-strand conformation polymorphism), TaqMan PCR, Loop-hybrid mobility shift assay, cycleave PCR, and PCR-RFLP^11^,^12^,^13^,^14^. To meet the more and more stringent clinical requests, high- or ultra-sensitive methods were enthusiastically developed including MALDI-TOF MS (matrix-assisted-laser-desorption-ionization time-of-flight mass spectrometry)^15^, PNA-LNA (peptide nucleic acid-locked nucleic acid) PCR clamp, Scorpions ARMS (amplified refractory mutation system), dHPLC (denaturing high performance liquid chromatography), single-molecule sequencing, and digital PCR-based or next generation sequencing (NGS)-based strategies^16^,^17^,^18^,^19^. CE-marked or FDA-approved assays are validated in reliability, traceability, procedure standardization, easily and popularly used in routine clinical service as companion diagnoses especially EGFR assays in international multicenter clinical trials. However, with the rapid growing of novel actionable and druggable candidates, the laboratory developed tests (LDT) have higher flexibility to meet the immediate clinical requests. Even the issue of well characterized quality assurance has come to a consensus, the guideline or the regulation is still debated. This was because of a lot of high-sensitive and high throughput platforms were developed and the difficulties in method validation needed to be solved. This study aimed to establish a customized EGFR mutation molecular testing by MALDI-TOF MS and to validate the characteristics of this platform for routine clinical practice. The first-line EGFR TKI was reimbursed by National Health Insurance (NHI) in Taiwan since June 2011. In this report, we have conducted the EGFR mutation companion diagnostics from Aug-2011 to Jul-2015 in Taiwan. We focused on the quality issues including method validation, procedure, turnaround time and statistical characteristics. This can be a reference for cancer molecular diagnostics.

## Results

### Procedure of Mutation Testing by MALDI-TOF MS

**Figure 1:**
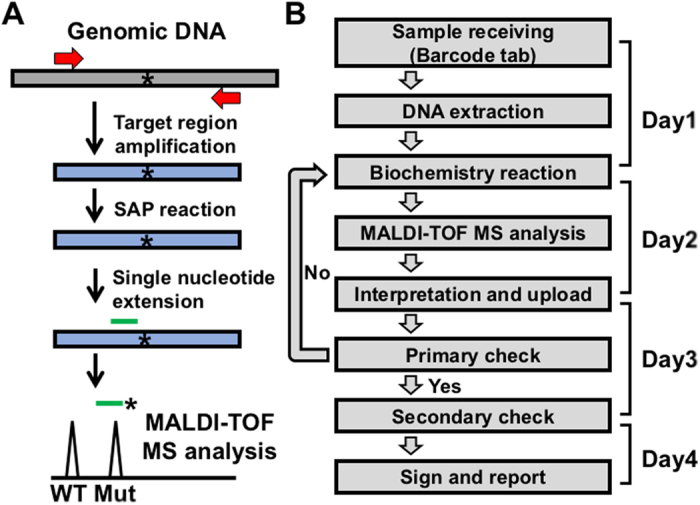
Schematic presentation of the principle of mutation detection by MALDI-TOF MS and the molecular testing process. (**A**) Genomic DNA extracted from samples was amplified by PCR primers. After inactivation of dNTP by SAP treatment, the target site-containing amplicons were further performed single nucleotide extension by the probe annealing to the nucleotide before the mutation site and ddNTP. The mutation specific product can be distinguished from the wild-type one in the mass spectrometry due to the incorporated nucleotide. (**B**) The procedure of molecular diagnostics can be completed within four working days starting with the sample receiving until data reported. dNTP, deoxynucleotide; ddNTP, dideoxynucleotide; SAP, shrimp alkaline phosphatase; PCR, polymerase chain reaction.

To establish routine clinical EGFR genetic testing in lung cancer patients, the pipeline of testing was firstly constructed (Fig. 1A). The experimental procedure was started from genomic DNA extraction from samples followed by PCR-based target amplification. After inactivation of dNTP by shrimp alkaline phosphatase (SAP) treatment, the single nucleotide extension reaction was performed by the specific probe annealed to one nucleotide before the mutation site. The incorporated ddNTP was different in the wild-type and mutant allele and the final products were further analyzed by MALDI-TOF MS. The mutation specific products can be distinguished from the wild-type ones in the spectrum due to the different molecular weights. The EGFR genetic testing was performed by the Pharmacogenomics Lab funded by National Research Program for Biopharmaceuticals (NRPB) Taiwan. All standard operation procedures were certified by ISO15189 regulation (Medical Laboratory 2695, No. L2695-140527). The testing process including sample receiving, testing and reporting was taken about four working days (Fig. 1B). Day 1 was initiated from sample receiving and unique barcode tabbing followed by nucleic acid extraction. Most of cases were continued to parts of biochemistry reactions. The biochemistry reactions included PCR, SAP reaction and single nucleotide extension. The reactions were ended at day 2 followed by MALDI-TOF MS analysis and data interpretation. At Day 3, the result was primary checked by laboratory scientists including quality control. After that, the final report was signed by two medical technologists at day 4. All cases adjudged to be needed confirmation at primary check will be repeated the testing procedure from biochemistry reaction.

### Quantification of EGFR Mutations Determined by MALDI-TOF MS

MALDI-TOF MS is a multi-function and flexible platform for gene testing. The major advantages are high sensitivity, low DNA quality requirement, capable of multiplex gene testing and quantification of mutation frequency. The principle of mutation quantification was shown in Supplementary Fig. 1A. The mutant allele competes with the wild-type allele for binding to detection probes. The ratio of mutant to wild-type signal height was calculated and reflected the percentage of mutant alleles among all alleles in the tested samples. To optimize the operation procedures of MALDI-TOF MS, the gDNAs from PBMC of healthy individuals and the DNAs from two well-established lung adenocarcinoma cell lines in which H1975 harbors both EGFR L858R and T790M mutations and PC9 harbors Del19 mutation were subjected to test as reference materials. The clear reproducible signals were obtained from MALDI-TOF MS for all control samples (Supplementary Fig. 1B). In EGFR L858R, Del19 and T790M detection, PBMC had no mutation signal while H1975 and PC9 showed the L858R/T790M mutation signals and the Del19 mutation signal, respectively. To test the repeatability and reproducibility, the calculated EGFR mutation frequency by MALDI-TOF MS was used as an index. Each control sample from 30 independent inspections was collected for variation analysis (Supplementary Fig. 1C). Among these mutations, PBMC had a background mutation frequency in L858R (0.0 ± 0.0), T790M (0.3 ± 0.3) and Del19 (1.4 ± 0.5). H1975 had high mutation frequency in L858R (67.4 ± 2.0), T790M (73.0 ± 1.2) but low mutation frequency in Del19 (1.3 ± 0.4). PC9 only had high mutation frequency in Del19 (87.1 ± 2.2) and low mutation frequency in L858R (0.0 ± 0.0) and T790M (0.3 ± 0.5). The coefficient of variation (CV) of mutation frequency were 2.98% for L858R (in H1975), 1.66% for T790M (in H1975) and 2.56% for Del19 (in PC9) respectively. These results suggested that MALDI-TOF MS can quantitatively and reproducibly detect EGFR mutations for clinical practice.

### Precision, Reference Range and Limit of Detection

**Figure 2:**
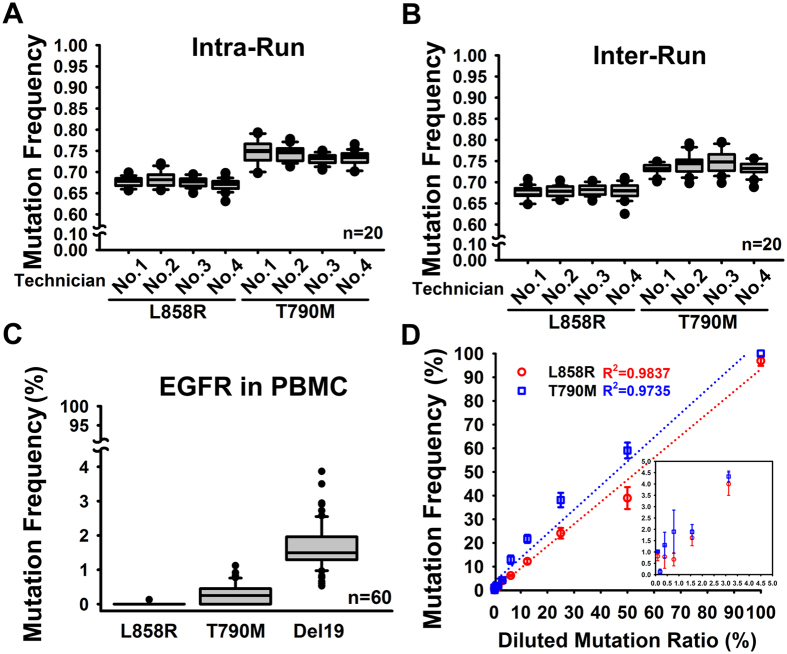
Precision, reference range and LOD of MALDI-TOF MS for EGFR mutation detection. (**A**) Intra-run precision test. Four independent technicians (No. 1~4) performed testing in 20 replicates by using the EGFR L858R/T790M harboring cells, H1975. The mutation frequencies were plotted in the box chart. (**B**) Inter-run precision test. Four independent technicians (No. 1~4) performed testing in 20 replicates in independent runs by using H1975 cells. The mutation frequencies were plotted in the box chart. (**C**) Reference range identification. Sixty PBMC samples were assessed for the EGFR mutation testing. The mutation frequencies were plotted in the box chart. (**D**) LOD of MALDI-TOF MS in the EGFR mutation detection. Correlation of theoretic dilution ratios and calculated mutation frequencies was calculated by linear regression analysis in EGFR L858R and T790M detections. Each dilution was assayed in triplicate. LOD, limit of detection.

To verify the analytic validity of MALDI-TOF MS platform we first performed the intra-run and inter-run precision test (Fig. 2A,B). In the intra-run test, the EGFR L858R and T790M mutations of H1975 cells were assayed by independent four technicians in 20 replicates independently. In total 80 replicates, the averaged mutation frequency of L858R was 67.66 ± 1.46% with 2.15% CV while T790M was 73.94 ± 2.05% with 2.77% CV (Fig. 2A and Supplementary Table 1). In the mention of technical variation, the CVs of 20 replicates by each technician were ranged from 1.65% to 2.68% in L858R and 1.67% to 3.69% in T790M. In the inter-run test, the averaged mutation frequency of L858R was 67.75 ± 2.37% with 3.50% CV while T790M was 73.86 ± 2.10% with 2.84% CV in total 80 replicates (Fig. 2B and Supplementary Table 1). For each technician, the CVs of 20 replicates were ranged from 1.91% to 5.87% in L858R and 1.76% to 3.29% in T790M. The scatter plot of L858R vs T790M mutation frequency in total 160 replicates from the intra-run and inter-run showed high performance of MALDI-TOF MS in precision (Supplementary Fig. 2). The evaluation of precision for Del19 was also performed by using PC9 cells (Del E746-A750 mutation) as a reference material (Supplementary Fig. 3). The CV for Del19 was 0.79% in the intra-run test while 1.50% in the inter-run test. In the mention of reference range determination, 60 genomic DNAs from PBMC of healthy individuals were utilized as normal samples (Fig. 2C). The result indicated that the EGFR mutation frequency in PBMC was 0.002 ± 0.016% (95% CI: 0.000–0.036) in L858R and 0.292 ± 0.289% (95% CI: 0.000–0.871) in T790M and 1.658 ± 0.625% in Del19 (95% CI: 0.000–2.961) (Supplementary Table 2). Limit of detection (LOD) for EGFR mutation was defined as the lowest percentage of mutant allele content among wild-type allele background. It was determined by the serial dilutions made by mixing the mutant EGFR plasmids with wild-type ones (Fig. 2D). Among totally constant 1000 plasmid copies, the correlation between theoretical diluted mutation ratio and MALDI-TOF MS calculated mutation frequency was plotted. The R^2^ of diluted mutation ratio versus mutation frequency was 0.9837 in L858R and 0.9735 in T790M. However, the confident quantification of mutation frequency was around 1% (Fig. 2D, inserted box).

### Accuracy Test, Analytical Sensitivity and Analytical Specificity

To address the accuracy of MALDI-TOF MS in EGFR mutation detection, we utilized the reference immortalized cell lines with naturally occurring disease-associated sequence variations or synthetic cloned DNA for testing according to the suggestion guideline^20^. All materials can be traced according to the information from quality documents, literatures, reference articles as well as database from bioresources (Table 1). In the double blind test, the EGFR mutation statuses including L858R, T790M and Del19 determined by MALDI-TOF MS were totally consistent with the statements in the public database. The artificial 50% mutant allele DNAs made up of the EGFR wild-type and L858R/T790M expression plasmids also exhibited the anticipated mutation frequency (Table 1 and Supplementary Fig. 4).

Analytical sensitivity and analytical specificity were tested by another set of 45 clinical FFPE samples (with sufficient amounts for quantitative DNA extraction, Supplementary Methods) from lung cancer patients and three PBMC samples. These samples were assessed for double blind EGFR mutation testing by traditional Sanger sequencing and MALDI-TOF MS methods in parallel (Table 2). None of EGFR L858R, T790M and Del19 was detected by both methods in three PBMC samples. Among the 45 FFPE samples, 14 had the L858R mutation and 9 had the Del19 mutation and one had L858R/T790M double mutations and 21 had no mutation. The results of MALDI-TOF MS were consistent with those of Sanger sequencing with 100% analytical sensitivity and 100% analytical specificity.

### Routine Testing Characteristics and Quality Monitoring

**Figure 3:**
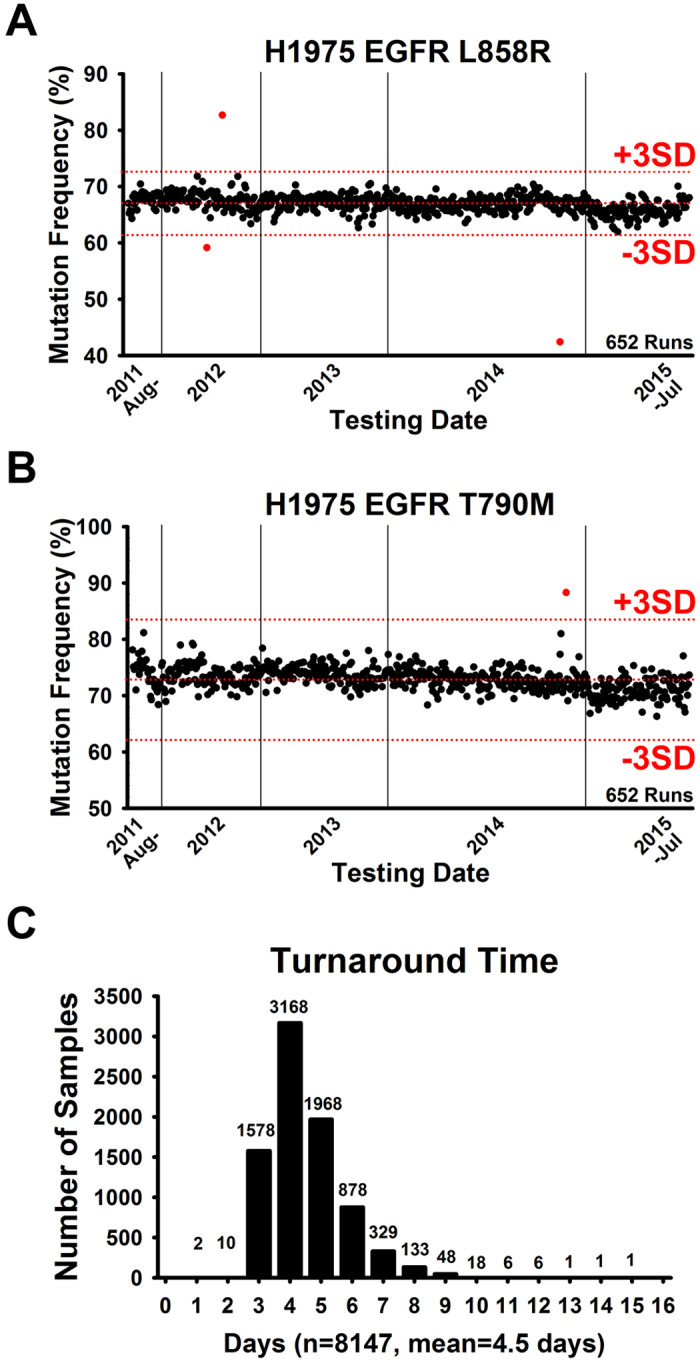
Quality monitor and turnaround time of MALDI-TOF MS. Levey-Jennings quality graph was used to monitor quality of MALDI-TOF MS by using DNA of H1975 cells from each run for the EGFR L858R mutation frequency (**A**) and the EGFR T790M mutation frequency (**B**). (**C**) The turnaround time of the EGFR mutation detection in 8,147 cases.

Since MALDI-TOF MS was established as the routine lung cancer molecular testing in Pharmacogenomics Lab, we analyzed totally 8,147 lung adenocarcinoma cases from Aug 2011 to Jul 2015 under ISO15189 regulation (Fig. 3 and Table 3). Among these cases, 4,299 cases were tested from Aug 2011 to Nov 2013 and parts of these (n = 1,772) had been reported in our previous study^21^. Additional 3,848 cases were tested from Dec 2013 to Jul 2015 by the same platform (additional 6,375 cases were included in this study). According to the statistical result, we analyzed 170 cases per month in average and 74.7% (n = 6,089) were FFPE samples (Table 3). Regarding to the testing fail rate, only 0.1% (n = 5) samples were fail in testing due to the poor DNA quality or reaction. Up to 94.6% (n = 7,708) of cases were reported at the first testing process while 5.3% samples (n = 434) were reported by further confirmation due to the inconsistence of replicates within one run (Table 3). Given EGFR L858R, T790M and Del19 mutations, the mutation prevalence were 24.7%, 3.8% and 23.1% respectively in tested cases similar with our previous study. The DNA concentrations from different sample types showed that all were various with a wide range. Pleura effusion and other sample types yielded related higher DNA concentration compared with other types (Supplementary Fig. 5). In each testing run, control materials including H1975 cell line harboring L858R/T790M and PBMC gDNA were assayed in parallel as a quality monitor of system. According to previous results, the H1975 cell line had stable mutation frequency and was suitable for systematic monitoring in the routine practice. Taking the mutation frequency of L858R or T790M in H1975 cells for Levey-Jennings quality graph, there were three L858R and one T790M tests out of 652 runs fail in quality monitoring (Fig. 3A,B). Starting from the sample receiving, we were in principle to report the data for clinical applicants in averaged 4.5 turnaround days (Fig. 3C).

## Discussion

Precision medicine points out that the treatment for individual cancer patient should consider their genetic information. Taking the advantage of new sequencing techniques and vast databases of information, the identification of potential actionable genetic aberrations is dramatically growing. On the other hand, this advance introduces an unprecedented revolutionary progress in laboratory practice. However, it has difficulties in the establishment of the standard operation procedure even consensus guidelines. According to the results from clinical trials, the prediction power of the molecular testing for therapeutic response was better than the traditional laboratory testing. The success of EGFR target therapy in lung cancer patients with EGFR mutations initiated the era of molecular diagnostics in cancer management^4^. In addition, the experience of prospective testing in Taiwan by Pharmacogenomics Lab can be a reference of cancer molecular testing in the future.

Even though we consider the precision issues including reproducibility (inter-run precision) and repeatability (intra-run precision) as well as the performance variations of technicians in this study, the accuracy is still a trouble due to the availability of reference materials. Herein, we utilized the traceable cell lines or synthetic DNAs as biomaterials to perform the accuracy testing according to recommendations^20^. It was noticed that occasionally EGFR mutations were detected in normal PBMCs with low mutation frequency (Fig. 2 and Supplementary Table 2). Based on the LOD established by the synthetic DNAs, the EGFR mutation frequency of PBMCs was lower than LOD. The data suggested that the low frequency found in PBMCs should be derived from the background noise of the assay. Characterization of the background is necessary for defining the cutoff value in routine practice. Furthermore, our results showed that the L858R mutation rate was 24.7% (2,012/8,147), Del19 mutation rate was 23.1% (1,884/8,147). The rate of overall EGFR activating mutations is consistent with the epidemiological statistics in Asian population^4^,^22^,^23^,^24^,^25^,^26^, this fact provides a robust clinical validation to prove the clinical utility of our system. To determine the analytical sensitivity and specificity of MALDI-TOF MS Sanger sequencing was acted as the gold standard method although the performance and successful rate of Sanger sequencing is largely limited in the poor DNAs or specimens. The main purpose of comparison between MALDI-TOF MS data and Sanger sequencing data is to perfect the analytic validity of MALDI-TOP MS not to investigate the limit of clinical specimen quality between both assays. Although the basic performance characteristics for Sanger sequencing had been mentioned, it still needed to consider whether the item of these characteristics should be concerned in the different mutation testing^27^. Furthermore, in our previous study have shown that some EGFR mutations of clinical specimens detected by a highly sensitive method cannot be identified by Sanger sequencing^15^.

Next, the TAT of molecular diagnostics was largely dependent on the methodology used, and the average TAT was around two weeks (10 working days)^28^. The averaged working time for sequencing-based assays particularly for the case of NGS was four to five working days indicated that the post-analytical data processing is time-intensive and complex^28^. Our system exhibited a relative short turnaround time (4.5 working days) (Fig. 3 and Table 3). Finally, the DNA concentration of extracted samples is an issue. In this study, the DNA concentration was varied with a wide range (Supplementary Fig. 5) which may be attributed by several confounding factors including the handling process of samples, the size of biopsy, the basic property of sample type, and the technical variation of extraction. In our routine practice, three 10μm thickness FFPE slices with over 0.5 cm-square tumor biopsy were recommended while the size with 2 mm cubic was recommended for fresh tissues.

In spite of growing up in sequencing-based or quantitative PCR-based detection platforms, more than 10 well-documented methods were used in EGFR mutation identification^29^. Recently, the emergence of NGS facilitated the high throughput and multiplex genetic testing in personalized medicine of cancers. Although the trend of NGS used in clinical molecular diagnostics was a consensus and authorized by US FDA, the risk-based regulatory framework was still a critical issue for quality assurance^30^.

Although the quality assurance of molecular diagnostics still had a lot of gray zone due to objective difficulties such as method validation, independent proficiency testing, and reference material availability, many guide lines and consensus agreements from the expert workgroups consist of experts were established^20^. According to CLIA (Clinical laboratory improvement amendment) regulations, the analytical validation should consider several characteristics such as precision, accuracy, analytical sensitivity, analytical specificity, reference range and reportable range as well as other relevant performance metrics^31^. In house or LDT assays used in cancer pharmacogenomics testing should follow such kinds of regulations. Although there was still a gray zone in method validation and quality system of molecular diagnostics, more and more consensuses from experts will form mature regulations^32^,^33^,^34^. In this study we demonstrated that our system including MALDI-TOF MS and the entire validation process is a convincing system and adheres to the consensus guidelines of CLIA. The clinical utility of our system is confirmed by more than 8,000 patients with lung adenocarcinoma since 2011 to 2015. The first-line TKIs for the EGFR mutation patients identified by our system were reimbursed by Taiwan NHI. The goal of this study is to provide an update on recent developments for advanced NSCLC patients with EGFR mutations characterized by actionable molecular or histological alterations. Taken together, the molecular diagnostics of cancer pharmacogenomics aimed to understand and identify the genetic aberrations that influence drug efficacy and cytotoxicity in cancer patients. The pipeline of molecular diagnostics in cancer pharmacogenomics has been widely executed in worldwide such as United States, France, Japan, China, Germany and Taiwan^9^,^21^,^35^,^36^,^37^. Each step of cancer pharmacogenomics study to prepare for the clinical routine practice including testing cohort selection, sample size optimization, phenotype consideration, statistical analysis, and validation needed to be carefully conducted^38^. The implementation required the cooperation between clinical physicians, pathologists, laboratory scientists and executive support. In conclusion, this study firstly provides the experience of an in-house molecular diagnostics system in cancer pharmacogenomics, especially EGFR mutations in lung cancer, from setup to routine practice and quality control in Taiwan.

## Methods

### Study cases

The 8,147 study cases were from a multicenter prospective observational trial approved by the Institutional Review Board of the participating institutes including IRB No. 201111039RIC (National Taiwan University Hospital Research Ethics Committee), IRB No. C08197 (Institutional Review Board of Taichung Veterans General Hospital), IRB No. DMR100-IRB-284(CR-2) (China Medical University and Hospital Research Ethics Committee), IRB No.CS12022 (Institutional Review Board of Chung Shan Medical University Hospital), and IRB No. REC102-7 (Taichung Tzu Chi Hospital Research Ethics Committee). Written informed consents for the genetic testing and clinical data records were obtained from all patients.

### Genomic DNA Extraction, EGFR Mutation Detection by Sanger Sequencing and MALDI-TOF MS

Genomic DNAs were extracted from the tumor samples by using QIAmp DNA Minikit (QIAGEN, CA) according to the manufacturer’s instruction. The mutation analysis of EGFR by Sanger sequencing has been described previously^39^. Detection and quantification of EGFR mutations by MALDI-TOF MS was described in our previous studies^15^,^40^. The method and the procedure were detailed in the supplementary material (see the supplementary material for additional details).

### Quality System

The testing performed in Pharmacogenomics Lab was under the regulation guideline of International Organization for Standardization (ISO) 15189. Pharmacogenomics Lab obtained ISO15189 certification from Taiwan Accreditation Foundation (TAF) since April-2013 (No. 2695). For external quality control, we participated proficiency test (PT) programs from College of American Pathologists (CAP) (Molecular Oncology, Program Code: EGFR) and European Molecular Genetics Quality Network (EMQN) (Program: lung cancer) twice a year since 2011.

### Method Validation

Materials used for MALDI-TOF MS method validation including cell lines and control DNAs were purchased or obtained from ATCC, NCI or other institutes (Table 1). The validation items included precision, accuracy, analytical sensitivity, analytical specificity and reference range. The strategy and the procedure were detailed in the supplementary material (see the supplementary material for additional details).

## Additional Information

**How to cite this article**: Su, K.-Y. *et al.* Implementation and Quality Control of Lung Cancer EGFR Genetic Testing by MALDI-TOF Mass Spectrometry in Taiwan Clinical Practice. *Sci. Rep.* **6**, 30944; doi: 10.1038/srep30944 (2016).

## Supplementary Material

Supplementary Information

## Figures and Tables

**Table 1:** Accuracy Test of MALDI-TOF MS in EGFR Mutation Testing by Traceable Biological Materials.

Material	Type	Source	EGFR Status	EGFR Typing by MALDI-TOF MS						Note
				L858R	(%)	T790M	(%)	Del19	(%)	
A549	Human lung carcinoma cell line	ATCC	WT	No	0.0	No	0.6	No	2.0	ATCC CCL-185
CpGenome^TM^	Genomic DNA controls	EMD Millpore	WT	No	0.0	No	0.3	No	2.1	EMD MILLPORE, Cat. S7822, U.S Patent#5,786,146
CL1-0	Human lung adenocarcinoma cell line	Lab home made	WT	No	0.0	No	0.6	No	2.4	Reference: Chu et. al.*
CL1-5	Human lung adenocarcinoma cell line	Lab home made	WT	No	0.0	No	0.1	No	2.2	Reference: Chu et. al.*
EKVX	Human lung adenocarcinoma cell line	NCI-60	WT	No	0.0	No	0.6	No	2.4	COSMIC ID: COSS905970
H1437	Human lung adenocarcinoma cell line	ATCC	WT	No	0.0	No	0.1	No	2.6	ATCC CRL-5872
HCC827	Human lung adenocarcinoma cell line	ATCC	Del19	No	0.0	No	0.1	Yes	95.5	ATCC CRL-2868
HCT116	Human colorectal carcinoma	ATCC	WT	No	0.0	No	0.2	No	2.6	ATCC CCL-247
HOP62	Human embryonic kidney cell line	NCI-60	WT	No	0.0	No	0.3	No	2.0	COSMIC ID: COSS905972
HOP92	Human embryonic kidney cell line	NCI-60	WT	No	0.0	No	0.3	No	2.2	COSMIC ID: COSS905973
HT29	Human colorectal adenocarcinoma	ATCC	WT	No	0.0	No	0.6	No	2.4	ATCC HTB-38
NCI-H1975	Human lung adenocarcinoma cell line	ATCC	L858R/T790M	Yes	66.3	Yes	73.8	No	2.5	ATCC CRL-5908
NCI-H226	Human lung squamous cell carcinoma cell line	ATCC	WT	No	0.0	No	0.1	No	2.5	ATCC CRL-5826
NCI-H322M	Human lung adenocarcinoma cell line	NCI-60	WT	No	0.0	No	0.2	No	2.3	COSMIC ID: COSS905967
NCI-H460	Human large cell lung carcinoma cell line	ATCC	WT	No	0.0	No	0.2	No	1.8	ATCC HTB-177
PC9	Human lung adenocarcinoma cell line	RIKEN BioResource Center	Del19	No	0.0	No	0.3	Yes	99.2	RIKEN BioResource ID: RCB4455
SW480	Human colorectal adenocarcinoma cell line	ATCC	WT	No	0.0	No	0.0	No	2.8	ATCC CCL-228
50% mutant plasmid	Cloned EGFR expression plasmid	Lab cloned	L858R/T790M	Yes	34.4	Yes	62.4	No	0.8	50% pcDNA3.1-EGFR L858R/T790M + 50% pcDNA3.1-EGFR WT

^*^Am J Respir Cell Mol Biol. 1997 Sep;17(3)353-60.

**Table 2:** Testing Concordance between MALDI-TOF MS and Sanger Sequencing.

Sample ID	Sample Type*	DNA Conc. (ng/μl)	EGFR Mutation Status**	
			MALDI-TOF MS	Sanger Sequencing
1	PBMC	1625.5	WT	WT
2	FFPE	540	L858R	L858R
3	FFPE	1128.3	WT	WT
4	FFPE	770.3	L858R	L858R
5	FFPE	767.1	WT	WT
6	FFPE	1552.5	L858R	L858R
7	FFPE	1327.7	WT	WT
8	FFPE	741.6	L858R	L858R
9	FFPE	736.4	WT	WT
10	FFPE	680.9	Del19	Del19
11	FFPE	573.4	WT	WT
12	FFPE	948.7	L858R	L858R
13	FFPE	898	WT	WT
14	FFPE	556.4	L858R	L858R
15	FFPE	548.2	WT	WT
16	FFPE	626.5	Del19	Del19
17	FFPE	1790	WT	WT
18	FFPE	1015.1	L858R	L858R
19	PBMC	2433	WT	WT
20	FFPE	595.9	Del19	Del19
21	PBMC	1754.3	WT	WT
22	FFPE	858	Del19	Del19
23	FFPE	337.2	WT	WT
24	FFPE	594.3	Del19	Del19
25	FFPE	971.5	L858R	L858R
26	FFPE	647.2	WT	WT
27	FFPE	485	Del19	Del19
28	FFPE	1277.5	WT	WT
29	FFPE	661.7	L858R	L858R
30	FFPE	557.6	WT	WT
31	FFPE	2208.7	L858R	L858R
32	FFPE	1108.3	WT	WT
33	FFPE	405.9	L858R	L858R
34	FFPE	772.7	WT	WT
35	FFPE	1010	Del19	Del19
36	FFPE	1719.1	WT	WT
37	FFPE	326.4	L858R+T790M	L858R+T790M
38	FFPE	194.2	WT	WT
39	FFPE	512.4	L858R	L858R
40	FFPE	313	WT	WT
41	FFPE	467.8	WT	WT
42	FFPE	734.5	Del19	Del19
43	FFPE	825.1	WT	WT
44	FFPE	907.3	L858R	L858R
45	FFPE	892.6	WT	WT
46	FFPE	619.2	L858R	L858R
47	FFPE	888.5	WT	WT
48	FFPE	1502.9	Del19	Del19

^*^FFPE, Formalin-fixed paraffin-embedded slices of tumor biopsy; PBMC, Peripheral blood mononuclear cell.

^**^Only EGFR L858R, T790M and Del19 Assayed.

**Table 3:** Summary of Molecular Testing Procedure Characteristics in Pharmacogenomics Lab.

	Number	(%)
***Testing Number***	8147	(100.0%)
Aug-Dec, 2011	690	(8.5%)
Jan-Dec, 2012	1688	(20.7%)
Jan-Dec, 2013	2119	(26.0%)
Jan-Dec, 2014	2417	(29.7%)
Jan-Jul, 2015	1233	(15.1%)
***Sample Type***	8147	(100.0%)
Extracted DNA	1175	(14.4%)
FFPE*	6089	(74.7%)
Pleural Effusion	665	(8.2%)
Fresh Tissue	187	(2.3%)
Other**	31	(0.4%)
***Result***	8147	(100.0%)
1st Run Reported	7708	(94.6%)
Repeated Reported	434	(5.3%)
Test Fail	5	(0.1%)
***EGFR Mutation Status***	8147	(100.0%)
L858R	2012	(24.7%)
T790M	311	(3.8%)
Exon19 Deletion	1884	(23.1%)
Others/Unfound***	4374	(53.7%)
***Average TAT*** (***days***)	4.5	
Aug-Dec, 2011	6.1	
Jan-Dec, 2012	4.3	
Jan-Dec, 2013	4.4	
Jan-Dec, 2014	4.3	
Jan-Jul, 2015	4.3	

^*^FFPE, Formalin-fixed paraffin –embedded.

^**^Others include pericardial effusion, cell pallets, ascites and CSF.

^***^Unfound represented samples without EGFR L858R, exon19 deletion and T790M mutations.
