# Supplementary material for: Implementation and Quality Control of Lung Cancer EGFR Genetic Testing by MALDI-TOF Mass Spectrometry in Taiwan Clinical Practice
Source: Sci Rep. 2016 Aug 2;6:30944. doi: 10.1038/srep30944 (PMC4969598; doi:10.1038/srep30944)
Supplement: Supplementary Information [file srep30944-s1.doc]

**Supplementary information**

**Implementation and Quality Control of Lung Cancer EGFR Genetic Testing by MALDI-TOF Mass Spectrometry in Taiwan Clinical Practice**

Kang-Yi Su, Jau-Tsuen Kao, Bing-Ching Ho, Gee-Cheng Chang, Chao-Chi Ho, Sung-Liang Yu

**Supplementary Methods**

**Genomic DNA Extraction, EGFR Mutation Detection by Sanger Sequence and MALDI-TOF MS**

Genomic DNA (gDNA) was extracted from the tumor samples by using QIAmp DNA Minikit (QIAGEN, CA) according to manufacturer’s instruction. The mutation analysis of EGFR genes by PCR direct sequencing has been described previously (1). The tyrosine kinase domain of the EGFR coding sequence, exons 19, 20 and 21 were amplified, purified and sequenced in an automatic ABI Prism 3100 DNA Analyzer. PCR amplicons were sequenced in both sense and antisense directions and analyzed by BLAST (basic local alignment search tool) against the cDNA sequence of the EGFR gene and the chromatograms were examined by three independent readers manually.

**EGFR Mutations Detection by MALDI-TOF MS**

Detection and quantification of EGFR mutations was described in our previous studies (2, 3). Briefly, the detection procedure was according to the user’s manual of the MassARRAY system (SEQUENOM, San Diego, CA). Extracted DNA was performed serial biochemical reactions including 40 cycles PCR reaction; SAP (shrimp alkaline phosphatase) treatment and 200 cycles signal nucleotide extension reaction. After SpectroClean Resin clean up, samples were loaded onto the matrix of SpectroCHIP by Nanodispenser (Matrix) then analyzed by Bruker Autoflex MALDI-TOF MS. Data were collected and analyzed by Typer4 software (SEQUENOM, San Diego, CA). The mutation frequency was calculated as %=(mutant-type height)/(mutant-type height+wild-type height) x 100. Height was obtained from Type4 software. For each sample, two to four replications were performed and the average of mutation frequencies was used for further analysis.

**Method Validation**

Materials used for MALDI-TOF MS method validation including cell lines and control DNAs were purchased or obtained from ATCC, NCI or other institutes (table 1). The validation items included precision, accuracy, analytical sensitivity, analytical specificity and reference range.

*Precision:* To test the precision, H1975 cell line harbored EGFR L858R/T790M double mutations, PC9 cell line harbored EGFR Del19, HCC827 cell line harbored EGFR Del19, and other cell lines without EGFR mutations including A549, CL1-0, CL1-5, EKVX, H1437, HCT116, HOP62, HOP92, HT29, NCI-H226, NCI-H322M, NCI-H460, and SW480 were utilized. To clarify the precision of intra-run and inter-run as well as personal variation, 4 technicians performed tests for 20 replicates independently.

*Accuracy:* We used cell lines, artificial synthetic DNA or traceable commercial control DNA that were naturally and stably with/without mutant variations as reference materials for accuracy testing.

*Analytical sensitivity and analytical specificity:* Analytical sensitivity and analytical specificity were tested by using gDNA from three PBMC samples and selected 45 formalin-fixed, paraffin-embedded (FFPE) samples which met the criteria of tumor content is greater than 75% assessed by pathologists routinely, at least 1cm x 1cm in area, and sufficient for 5 slices with 10μm thickness. All of them were assessed for EGFR mutation testing by traditional Sanger sequencing and MALDI-TOF MS in parallel and double blind. The analytical sensitivity and analytical specificity of MALDI-TOF MS were evaluated by using Sanger sequencing as a gold standard assay.

*Limit of Detection:* The serial dilution of wild-type and mutant-type EGFR expression plasmids constructed in pcDNA3.1-EGFR were used to LOD of MALDI-TOF MS. In total 1,000 input copies, the percentage of mutant EGFR plasmid was ranged from 100% to 0%. Each dilution was assessed by EGFR mutation testing and calculated for mutation frequency. The detection limitation of MALDI-TOF MS was defined as the mutation percentage of the lowest dilution fold while the height of signal peak can be detected by Type4 software.

*Reference range:* The reference range was defined as the spectrum of consensus non-EGFR mutation samples. We utilized 60 gDNAs from PBMC samples for EGFR L858R, T790M and Del19 mutation testing to identify non-mutation spectrum.

**Supplementary Figure Legends**

**Supplementary Fig. 1.** Quantitative EGFR mutation detection by MALDI-TOF MS. (A) Illustration of the principle of quantitative EGFR mutation detection. Mutant (Mut) allele and wild-type (WT) allele mutually compete detection probes (arrows) in a single nucleotide extension reaction. The incorporated single nucleotides (close triangle represents mutant site and close circle represents wild-type site) result in the corresponding peaks in spectrum based on the molecular weight and the intensity of each peak reflects the allele amount in starting materials. (B) The EGFR L858R, T790M and Del19 spectrums of control samples including PBMC genomic DNA (PBMC), H1975 and PC9 human lung cancer cell lines. The arrow indicates the signal from mutant allele. (C) Reproducibility analysis of MALDI-TOF MS. The EGFR L858R, T790M and Del19 mutation frequencies were calculated in control samples with 30 independent replications.

**Supplementary Fig. 2.** Scatter plot of precision test in EGFR L858R and T790M mutations. H1975 cell line was used for precision test in 160 replicates, 80 replicates for intra-run precision test and 80 replicates for inter-run precision test, by MALDI-TOF MS and the mutation frequencies of EGFR L858R and T790M were illustrated by scatter plot. The insertion box was magnification of 160 replicates.

**Supplementary Fig. 3.** Precision test of EGFR Del E746-A750 mutation in PC9 cell line by MALDI-TOF MS. Intra-run and inter-run precision test were performed in 20 replicates by using EGFR Del E740-A750 harbored PC9 cell line. The mutation frequency was plotted in the box chart.

**Supplementary Fig. 4.** Spectrums of EGFR L858R, T790M and Del19 mutations detected by MALDI-TOF MS in cell lines and artificial DNA mixture. Red arrows indicated the expected site where the mutant signal should be presented.

**Supplementary Fig. 5.** Box chart of DNA concentration of 8,147 cases from different sample types. The mean concentration is indicated on the top of each sample type and the case number of each sample type is labeled in the below. Others include pericardial effusion, cell pallets, ascites and CSF. FFPE, Formalin-fixed paraffin-embedded slices of tumor biopsy; PE, pleura effusion.

**References**

1. Shih JY, Gow CH, Yu CJ, Yang CH, Chang YL, Tsai MF, et al. Epidermal growth factor receptor mutations in needle biopsy/aspiration samples predict response to gefitinib therapy and survival of patients with advanced nonsmall cell lung cancer. Int J Cancer 2006;118:963-9.

2. Tsai TH, Su KY, Wu SG, Chang YL, Luo SC, Jan IS, et al. Rna is favourable for analysing egfr mutations in malignant pleural effusion of lung cancer. The European respiratory journal 2012;39:677-84.

3. Su KY, Chen HY, Li KC, Kuo ML, Yang JC, Chan WK, et al. Pretreatment epidermal growth factor receptor (egfr) t790m mutation predicts shorter egfr tyrosine kinase inhibitor response duration in patients with non-small-cell lung cancer. J Clin Oncol 2012;30:433-40.

**Supplementary T**ables

| **Supplementary Table 1.** Intra-Run and Inter-Run Precision Test of Detection Platform by H1975 Reference Cell Line | | | | | | | |
| --- | --- | --- | --- | --- | --- | --- | --- |
|  | **EGFR L858R** | | |  | **EGFR T790M** | | |
|  | Measured mutation frequency (%) | CV (%) | N |  | Measured mutation frequency (%) | CV (%) | N |
|  |  |  |  |  |  |  |  |
| Intra-Run* | 67.66±1.46 | 2.15 | 80 |  | 73.94±2.05 | 2.77 | 80 |
| Technician |  |  |  |  |  |  |  |
| No.1 | 67.75±1.12 | 1.65 | 20 |  | 74.78±2.76 | 3.69 | 20 |
| No.2 | 68.23±1.83 | 2.68 | 20 |  | 74.48±1.87 | 2.51 | 20 |
| No.3 | 67.64±1.16 | 1.71 | 20 |  | 73.15±1.22 | 1.67 | 20 |
| No.4 | 67.03±1.45 | 2.16 | 20 |  | 73.36±1.67 | 2.28 | 20 |
|  |  |  |  |  |  |  |  |
| Inter-Run** | 67.75±2.37 | 3.50 | 80 |  | 73.86±2.10 | 2.84 | 80 |
| Technician |  |  |  |  |  |  |  |
| No.1 | 67.07±3.93 | 5.87 | 20 |  | 73.14±1.29 | 1.76 | 20 |
| No.2 | 67.82±1.30 | 1.92 | 20 |  | 74.31±2.35 | 3.17 | 20 |
| No.3 | 68.22±1.30 | 1.91 | 20 |  | 74.74±2.46 | 3.29 | 20 |
| No.4 | 67.88±1.94 | 2.86 | 20 |  | 73.23±1.74 | 2.38 | 20 |

*Intra-Run indicated EGFR L858R and T790M mutation frequency of H1975 cell line were assayed for 20 replicates by each personnel (No.1-No.4) within the same run.

**Inter-Run indicated EGFR L858R and T790M mutation frequency of H1975 cell line were assayed for 20 replicates by each personnel (No.1-No.4) within 20 different runs.

| **Supplementary Table 2.** EGFR Mutation Frequency in Normal PBMC for Reference Range Estimation | | | | | |
| --- | --- | --- | --- | --- | --- |
|  | Mutation Frequency (%) | 1SD | 2SD | 3SD | 95% CI |
| EGFR (n=60) |  |  |  |  |  |
| L858R | 0.002 | 0.016 | 0.033 | 0.050 | 0.000-0.036 |
| T790M | 0.292 | 0.289 | 0.579 | 0.868 | 0.000-0.871 |
| Del19 | 1.658 | 0.625 | 1.304 | 1.955 | 0.000-2.961 |

**Supplemental Figures**

**Supplementary Fig. 1.**

**
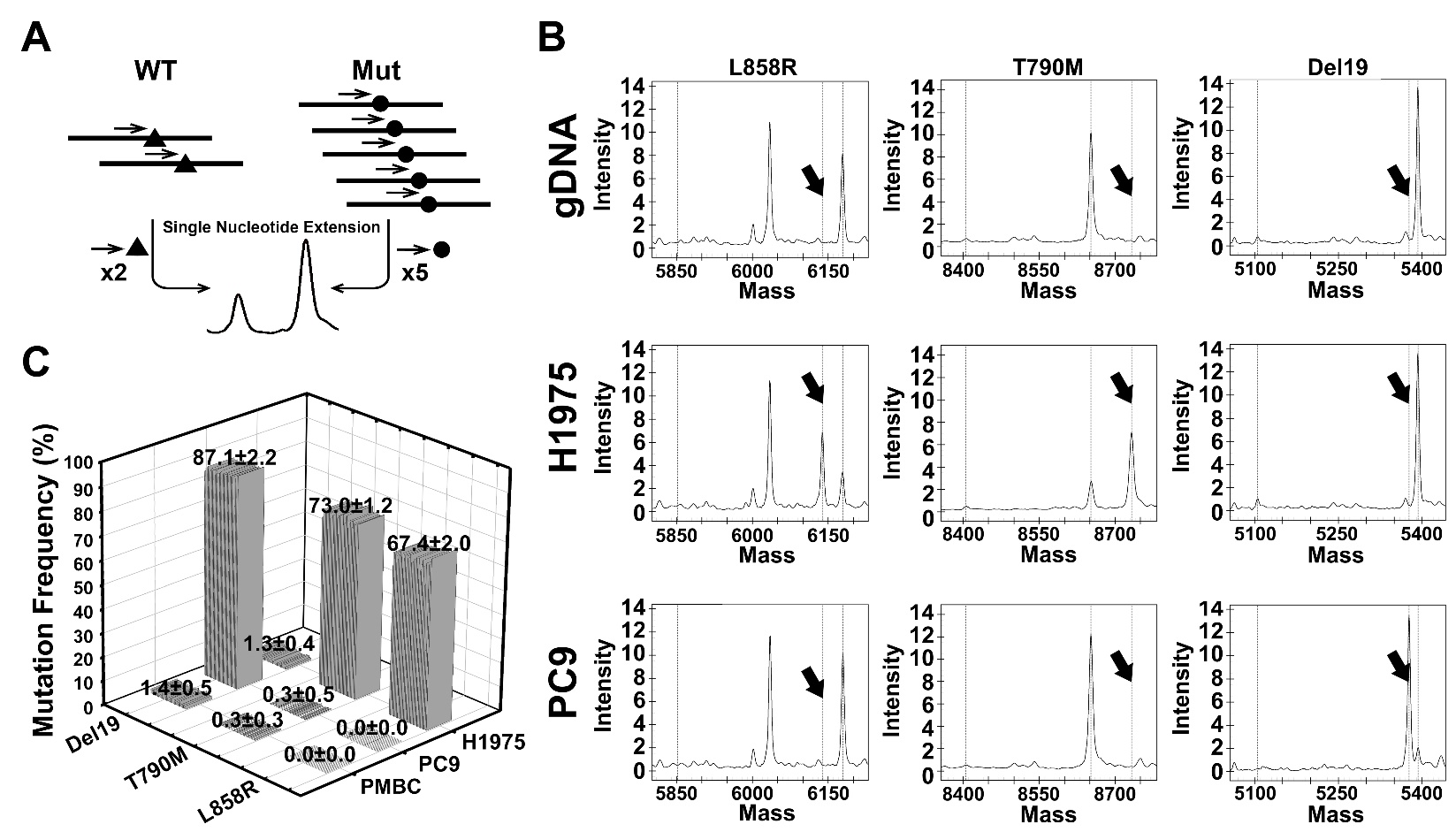
**

**Supplementary Fig. 2.**

**
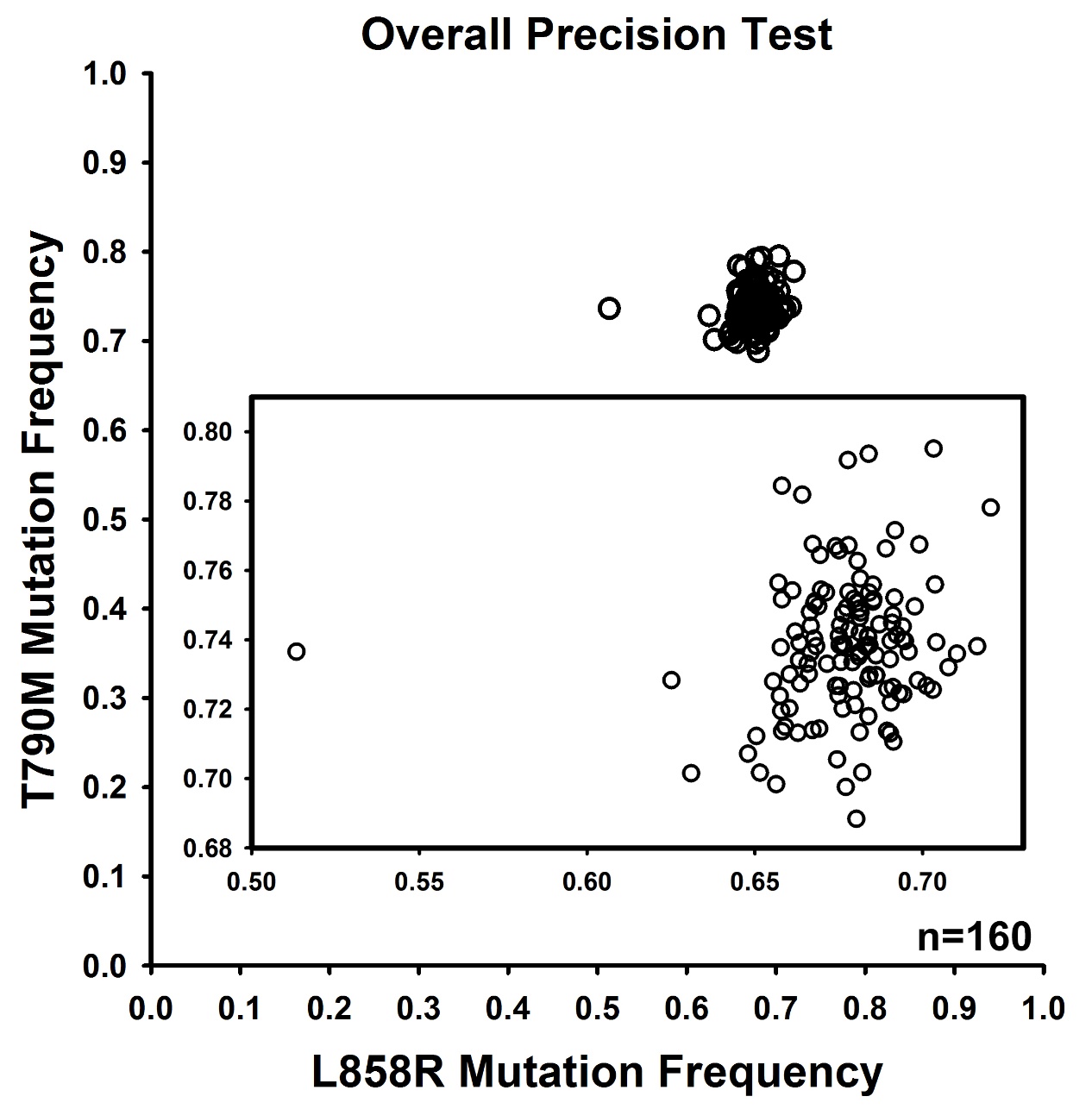
**

**Supplementary Fig. 3**

**
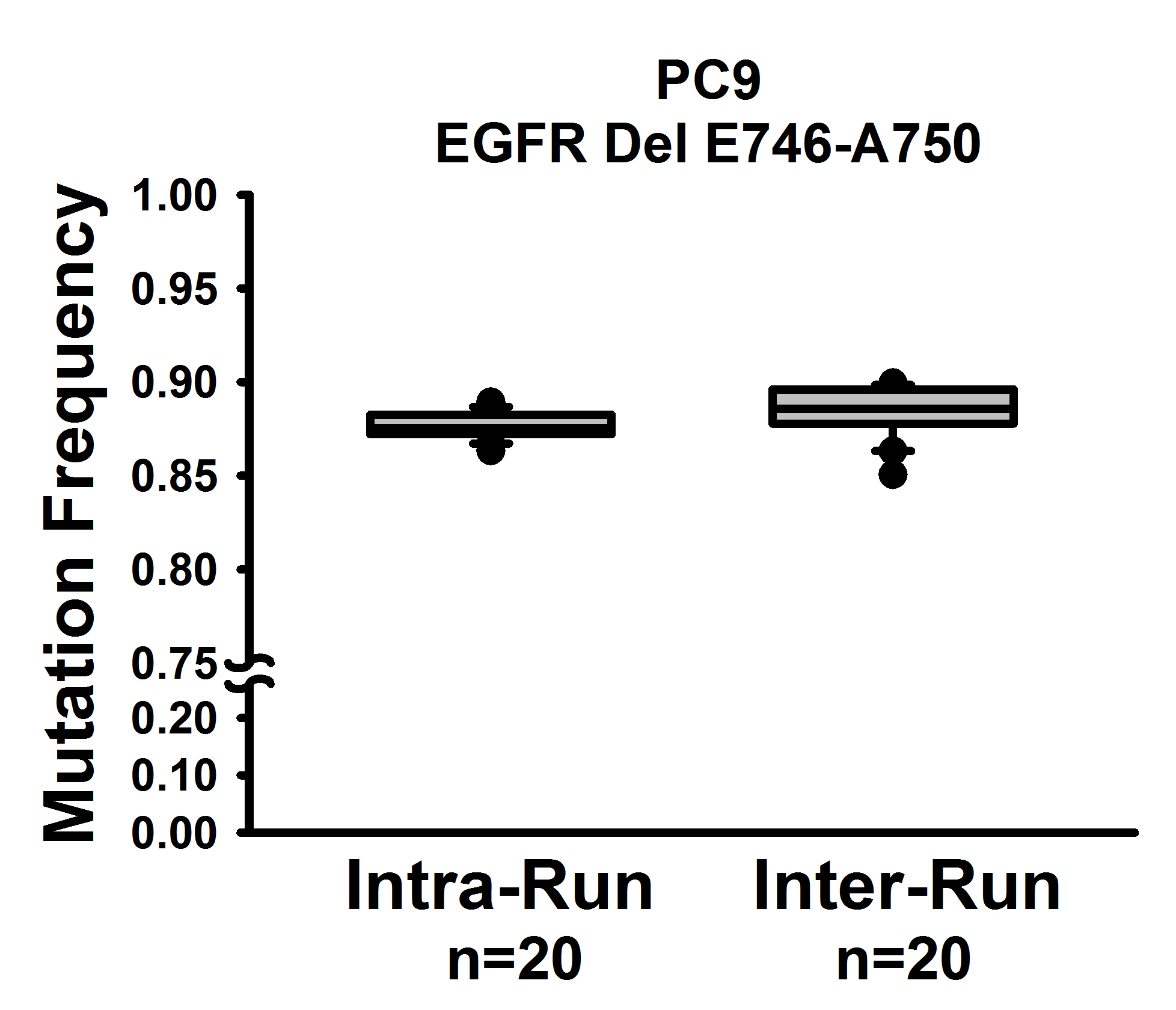
**

**Supplementary Fig. 4**

**
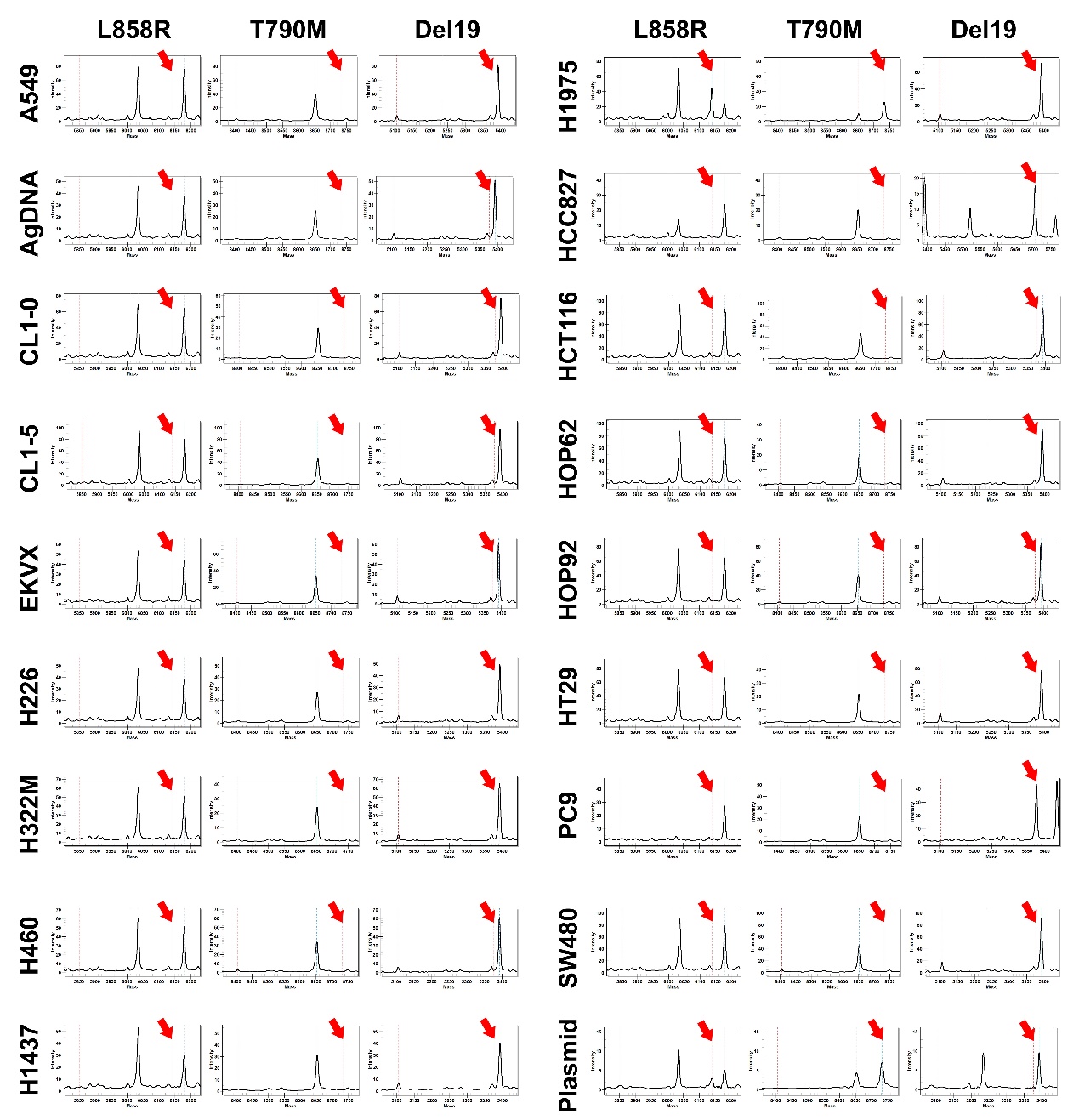
**

**Supplementary Fig. 5**

**
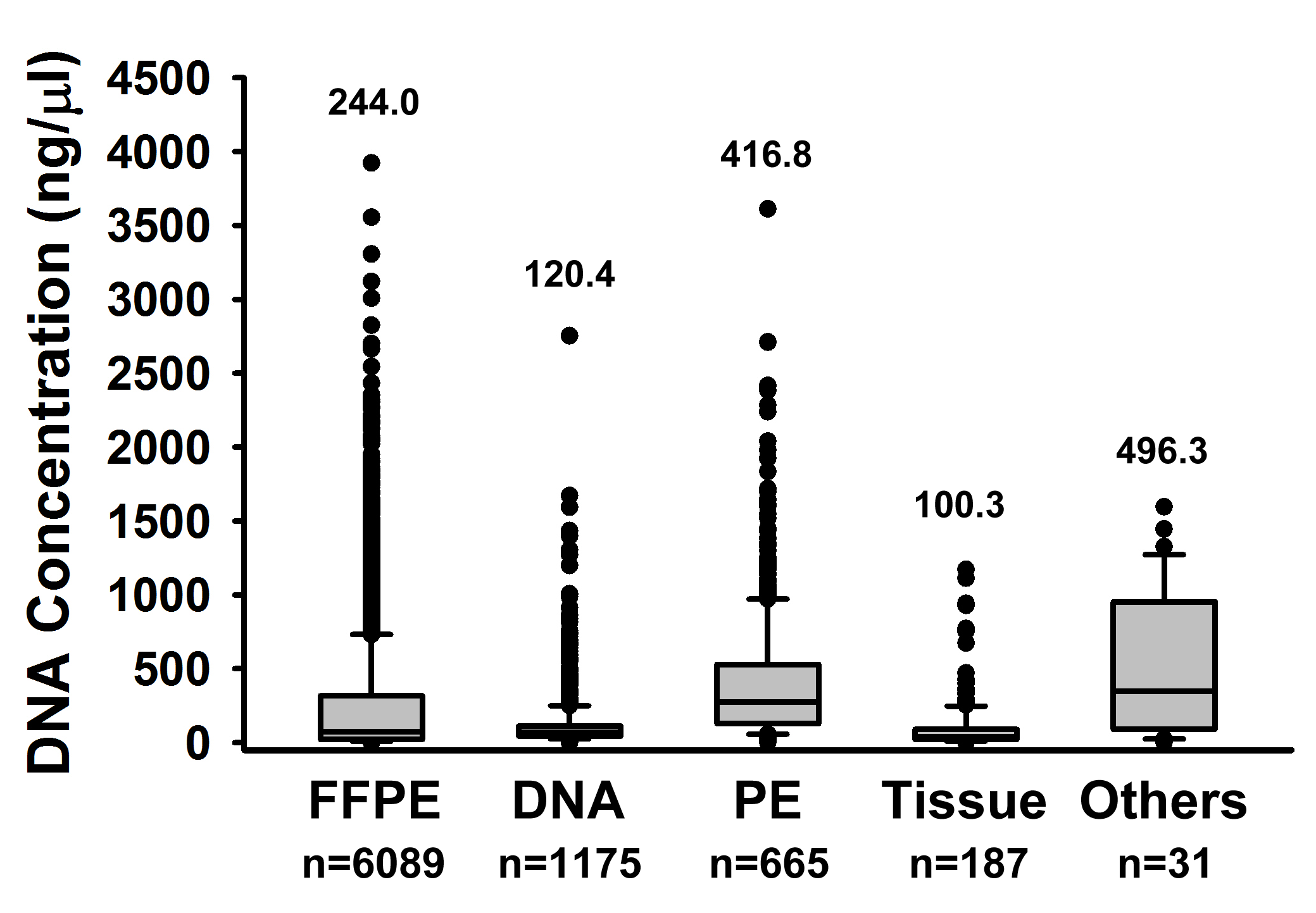
**
